# LncRNA SNHG16 drives proliferation, migration, and invasion of hemangioma endothelial cell through modulation of miR‐520d‐3p/STAT3 axis

**DOI:** 10.1002/cam4.1562

**Published:** 2018-05-29

**Authors:** Wenke Zhao, Hao Fu, Shasha Zhang, Shengkai Sun, Yang Liu

**Affiliations:** ^1^ Xinxiang Medical University Henan China; ^2^ The Affiliated Hospital of Logistics University of PAP Tianjin China; ^3^ General Hospital of Air Force, PLA Beijing China; ^4^ Shanghai Fourth People's Hospital Shanghai China

**Keywords:** hemangioma, miR‐520d‐3p, proliferation, SNHG16, STAT3

## Abstract

It has been verified that long noncoding RNAs (lncRNAs) have great effects on various biological behaviors of human diseases. Although more and more lncRNAs have been studied in human cancers, countless lncRNAs still need to be excavated. This study aims to investigate the impacts of lncRNA SNHG16 on proliferation and metastasis of human hemangioma endothelial cell (HemECs). qRT‐PCR analysis was carried out to explore the expression pattern of SNHG16, miR‐520d‐3p, and STAT3. The effect of SNHG16 on cell proliferation was detected by MTT and colony formation assay. Flow cytometry analysis was performed to test the apoptosis of HemECs cells. Migration and invasion of HemECs cells were determined and examined by transwell assays. Tube formation assay helped to observe the influence of SNHG16 expression on the vasoformation of HemECs cells. The correlations among SNHG16, miR‐520d‐3p, and STAT3 were certified by bioinformatics analysis, pull‐down assay, and dual‐luciferase reporter assay. Finally, rescue assays were conducted to demonstrate the effects of SNHG16‐miR‐520d‐3p‐STAT3 axis on biological behaviors of HemECs cell. SNHG16 was strongly expressed in proliferating phase hemangioma tissues and HemECs cells. Silenced SNHG16 negatively affected proliferation, migration, and invasion of HemECs cell. LncRNA SNHG16 acted as a ceRNA to upregulate STAT3 through binding with miR‐520d‐3p in HemECs cell. LncRNA SNHG16 acted as a ceRNA to drive proliferation, vasoformation, migration, and invasion of HemECs cells through modulating miR‐520d‐3p/STAT3 axis.

## 1 INTRODUCTION

Hemangioma (HA) is a kind of neoplasm which commonly occurs in the soft tissue of young children. 75%‐80% of HA patients are females.^1^ This disease often occurs when a child was born or after 2 weeks. HA threatens the lives of young children for its high speed in growth or invasion.^2^, ^3^, ^4^, ^5^ Therefore, it is urgent to do more research in hemangioma.

It is commonly acknowledged that long noncoding RNAs (lncRNAs) are a group of transcripts without ability to code proteins. LncRNAs are longer than 200 nt and constitute a large quantity (about 4%‐9%) of mammalian transcriptomes.^6^, ^7^ Previous studies have certified that lncRNAs almost participated in all aspects of biological behaviors, such as epigenetics, transcription, and post‐transcription. Additionally, they are proved to be involved in biological processes both physiologically and pathologically. Moreover, lncRNAs can modulate cell growth and differentiation, reprogramming, and stress response.^8^, ^9^, ^10^, ^11^ The specific molecular mechanisms of lncRNAs enable them to affect various biological processes. LncRNAs may directly bind with some particular DNA strands or RNA strands to modulate transcription or translation. Furthermore, they may form the functional complex through recruiting RNAs and proteins in cytoplasm or nucleus.^12^ ceRNA is a common role that is played by lncRNA. ceRNA model is commonly regarded as the functional medium between lncRNAs and mRNAs which are interacted with the same miRNAs.^13^ In this study, we hypothesized that SNHG16 acted as a ceRNA to upregulate STAT3 by binding with miR‐520d‐3p in HemECs cells.

snoRNA host gene 16 has been verified to be observably upregulated in human malignant carcinomas. It exerted oncogenic functions in various human cancers. For example, lncRNA SNHG16 improved tumorigenesis of bladder cancer via epigenetically silencing P21,^14^ SNHG16 acts as a ceRNA to promote cervical cancer progression,^15^ SNHG16 was an unfavorable prognostic factor in gastric cancer.^16^ All these previous studies showed that upregulation of SNHG16 is an oncogenic element in carcinogenesis. Despite the previous studies have revealed the molecular mechanism and biological function of SNHG16 in cancers, the specific role of it in hemangioma is still a secret. This study aimed to study the influences of dysregulated SNHG16 on biological behaviors of hemangioma endothelial cell.

## 2 MATERIALS AND METHODS

### 2.1 Tissue samples

Normal skin tissues (n = 12), involuting phase HAs (n = 12), and proliferating phase HAs (n = 12) were collected from patients who were diagnosed with hemangioma in The Affiliated Hospital of Logistics University of PAP. Each patient had signed the informed consent before this study. This study had obtained the approval of the ethics committee of The Affiliated Hospital of Logistics University of PAP.

### 2.2 Cell culture and transfection

HemECs cells were obtained from Shanghai Outdo Biotech Co. (Shanghai, China). Cells were preserved and maintained in Endothelial Basal Medium‐2 (EBM‐2; Cambrex Bio Science, Walkersville, MD, USA) which containing 10% FBS (Invitrogen, Shanghai, China).

To knock down SNHG16 in HemECs cells, the specific shRNAs and NC‐shRNA (sh‐NC) were purchased from Santa Cruz Biotechnology Inc. (Dallas, TX, USA), whereas STAT3 was overexpressed using pcDNA 3.1 vector (RiboBio, Guangzhou, Guangdong, China). Additionally, miR‐520d‐3p mimics and inhibitors were also synthesized by RiboBio to overexpress or silence miR‐520d‐3p.

### 2.3 RNA isolation and qRT‐PCR

At first, we isolated total RNAs with TRIzol reagent (Invitrogen, Grand Island, NY). Reverse transcription was finished using Superscript III transcriptase (Invitrogen, Grand Island, NY). qRT‐PCR was conducted in a Bio‐Rad CFX96 system in which a SYBR green was used to detect the mRNA level of gene. PCRs were conducted under the conditions as follows: 50°C for 2 minutes, 95°C for 8.5 minutes, followed by 45 cycles for about 15 seconds at 95°C and 60°C for 1 minute. The extension is 95°C for 1 minute, 55°C for 1 minute, and 55°C for 10 seconds. To clearly figure out the expression of genes in this study, GAPDH was seen as a control. All experimental steps were repeated for more than two times.

### 2.4 Cell proliferation assay

Cells were placed in 24‐well plates at a concentration of 3000 cells per well. Then, they were cultured for about 48 hours. Next, MTT regent was applied to replace media. DMSO was utilized to dissolve the blue crystals. At length, cell viability was identified by measuring the absorbance at 570 nm. All experimental samples were made in triplicate.

For colony formation assay, cells were plated in six‐well plates at a density of 3000 cells per well. They were then incubated in DMEM (containing 10% FBS) at 37°C. Two weeks later, cells were washed by PBS and fixed by methanol for 0.5 hours. After fixed dots were stained by 1% crystal violet, the number of colonies was manually calculated. All experimental samples were made in triplicate.

### 2.5 Cell migration and invasion assay

Firstly, cells were put into a 6‐well plate after necessary treatments and incubated for 3 days. We coated the upper chambers with Matrigel (1:20, BD Corning) for about 2 hours before the cells were plated. Next, we collected cells with serum‐free media and plated them (1 × 10^5^/mL) into the upper chambers. Then, 750 μL medium with 10% FBS was added into the lower chambers followed by incubation for 12 hours at 37°C with 5% CO_2_. Finally, we found that the invaded cells permeated methanol; thus, we stained them with 0.1% crystal violet in a dark room. All experimental samples were made in triplicate.

To calculate cell migration ability, we conducted transwell assay too. We performed this assay in accordance with the experimental method of a previous study.^17^ All experimental samples were made in triplicate.

### 2.6 Flow cytometry analysis

Cells were transfected with sh‐SNHG16 and sh‐NC. Two days later, they were harvested. Flow cytometry analyses measured apoptosis rate by using Annexin V: FITC Apoptosis Detection Kits (BD Biosciences, USA), following the user guide. All samples were made in triplicate.

### 2.7 Endothelial tube formation assay

Before transfection, HemECs cells were seeded on the 96‐well plates and incubated in an atmosphere containing 5% CO_2_ for 6 hours at 37°C. Matrigel (BD Biosciences, San Jose, CA, USA) and ECM were mixed at a ratio of 1:2. Next, each well of 96‐well plates was added into 50 μL of the mixture. They were then incubated for 20 minutes at 37°C for Matrigel solidification. Tube formation was pictured under a microscope and quantified by Image J software. All experimental samples were made in triplicate.

### 2.8 RNA FISH

RNA FISH KIT was bought from RiboBio (Guangzhou, China). The RNA FISH probe mix for SNHG16, 18S, or U6 was synthesized and produced by RiboBio. RNA FISH was conducted as previously reported.^18^ DAPI (RiboBio) was used to counterstain nuclei. A laser scanning confocal microscope was used to capture the high resolution (ZEISS, Jena, Germany).

### 2.9 Subcellular fraction assay

PARIS^™^ Kit (Ambion, Austin, TX) was utilized for the nuclear fractionation. Before this experiment was conducted, we collected cells (1 × 10^7^) and suspended them again in the cell fraction buffer. For next experimental procedures, cells were incubated the treated on ice for 10 minutes. After the centrifugation was finished, we discarded the upper solution and preserved the nuclear pellet to extract RNA using a cell disruption buffer. All experimental samples were made in triplicate.

### 2.10 RIP assay

Based on the user guide, EZMagna RIP kit (Millipore, Billerica, MA, USA) was utilized for RNA immunoprecipitation. We scraped off HemECs cells from the plates and lysed in 100% RIP lysis buffer, after which cell extract was preserved in RIP buffer which containing magnetic beads absorbed human anti‐Ago2 antibody (Millipore). Normal mouse IgG (Millipore) was the NC. Moreover, the concentration of RNA was assessed by the use of a NanoDrop spectrophotometer (Thermo Scientific), while the quality of RNA was evaluated using a bioanalyzer (Agilent, Santa Clara, CA, USA). Finally, qRT‐PCR analysis helped us analyzed the purified RNA. All experimental samples were made in triplicate.

### 2.11 Pull‐down assay

miR‐520d‐3p, miR‐520d‐3p‐Mut, and miR‐520d‐3p‐NC were biotinylated to be Bio‐miR‐520d‐3p‐WT, Bio‐520d‐3p‐MUT, and Bio‐NC by GenePharma Company (Shanghai, China). They were then transfected into HemECs cells. Four‐eight hours later, cells were collected and lysed. They were then incubated with Dynabeads M‐280 Streptavidin (Invitrogen, CA) for 10 minutes and washed with buffer. The bound RNAs were quantified and analyzed by qRT‐PCR.

### 2.12 Luciferase reporter assay

Firstly, HemECs cells were cotransfected with pmirGLO‐SNHG16‐wt or pmirGLO‐SNHG16‐mut (Sangon). Also, Lipofectamine 2000 (Invitrogen, Carlsbad, CA, USA) was utilized to transfect with miR‐520d‐3p mimics or miR‐NC. The relative luciferase activity was actually measured with a dual‐luciferase reporter assay kit (Promega) when cells were transfected for 48 hours. Renilla luciferase activity was taken as the NC to normalize the relative luciferase activity.

### 2.13 Western blot assay

Firstly, proteins were isolated with lysis buffer and electrophoretically transferred onto PVDF membranes (Millipore, Billerica, MA). Then we covered the membranes with bovine serum albumin (Sigma‐Aldrich, St. Louis, MO). The next step is to treat with primary antibodies all night at 4°C. Thereafter, incubation of secondary antibodies was conducted at normal temperature for 1 hour. The primary antibodies were shown as follows: cleaved caspase 3 (ab2302; 1:2000 dilution) cleaved caspase 9 (ab2324; 1:2000 dilution), anti‐STAT3 (ab119352; 1:2000 dilution), and snit‐GAPDH (ab8245; 1: 3000 dilution). The secondary antibody was Rabbit Anti‐Mouse IgG H&L (ab6728; 1: 2000 dilution). In order to visualize protein bands, an ECL chemiluminescent detection system (Thermo Fisher Scientific, Rochester, NY) was utilized. Finally, all proteins were exposed to X‐ray film. All experimental procedures were repeated for more than two times. All antibodies used in this experiment were bought from Abcam (USA).

### 2.14 Statistical analysis

SPSS v.17.0 (SPSS, Chicago, IL) was used for statistical analyses. Data represent means ± SD were obtained from more than two experiments. Differences between the two groups were analyzed with Student's *t* test, whereas multiple comparisons were made using one‐way ANOVA. Correlations among SNHG16, miR‐520d‐3p, and STAT3 were analyzed using Spearman's correlation analysis. Data were considered statistically significant when *P* < .05.

## 3 Results

### 3.1 The effects of silenced SNHG16 on proliferation and apoptosis of HemECs cells

**Figure 1:**
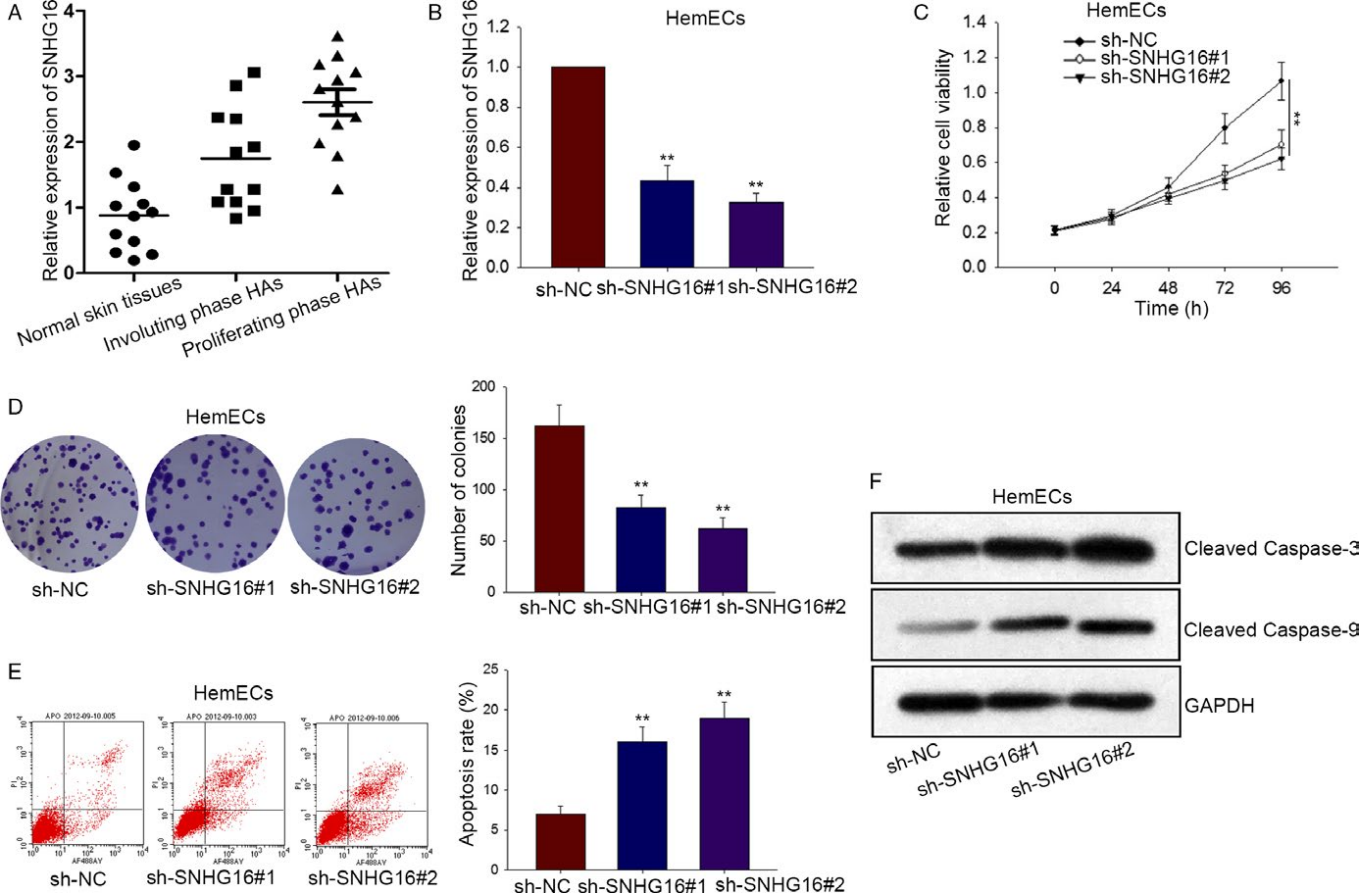
The effects of silenced SNHG16 on proliferation and apoptosis of HemECs cells. A, The expression of SNHG16 was tested by qRT‐PCR in three different types of tissues. B, The sh‐SNHG16 and sh‐NC were transfected, respectively, into HemECs for loss‐of‐function assay. C‐D, MTT assay and colony formation assay examined cell proliferation in sh‐NC and sh‐SNHG16 groups. E, Cell apoptosis was probed by flow cytometry analysis after shRNA transfection. F, The levels of apoptotic proteins were tested in HemECs cell by Western blot analysis. Data were acquired from multiple experiments for mean ± SD. *P < .05, **P < .01 compared with controls

To investigate the biological role of SNHG16 in hemangioma, the expression of SNHG16 was firstly examined in three different types of tissues (Figure 1A). The result suggested that the levels SNHG16 expression were obviously higher in proliferating phase HA tissues than that in involuting phase HA tissues and normal tissues. Next, SNHG16 was downregulated in HemECs cell by transfecting with shRNA (Figure 1B). In order to observe the impacts of silenced SNHG16 on proliferation, MTT and colony formation assay were performed in HemECs cell. According to the result of MTT assay, we knew that cell viability of HemECs was greatly inhibited by sh‐SNHG16 (Figure 1C). Similarly, silenced SNHG16 could negatively modulate colony formation rate of HemECs cell (Figure 1D). Moreover, cell apoptosis was examined by flow cytometry analysis and Western blot analysis. As illustrated in Figure 1E, cell apoptosis was promoted and accelerated by silenced SNHG16. The levels of apoptotic proteins (Caspase‐3 and Caspase‐9) were enhanced by sh‐SNHG16 (Figure 1F). Therefore, we concluded that silenced SNHG16 repressed cell proliferation and motivated cell apoptosis.

### 3.2 The influences of silenced SNHG16 on migration, invasion, and vasoformation of HemECs cells

**Figure 2:**
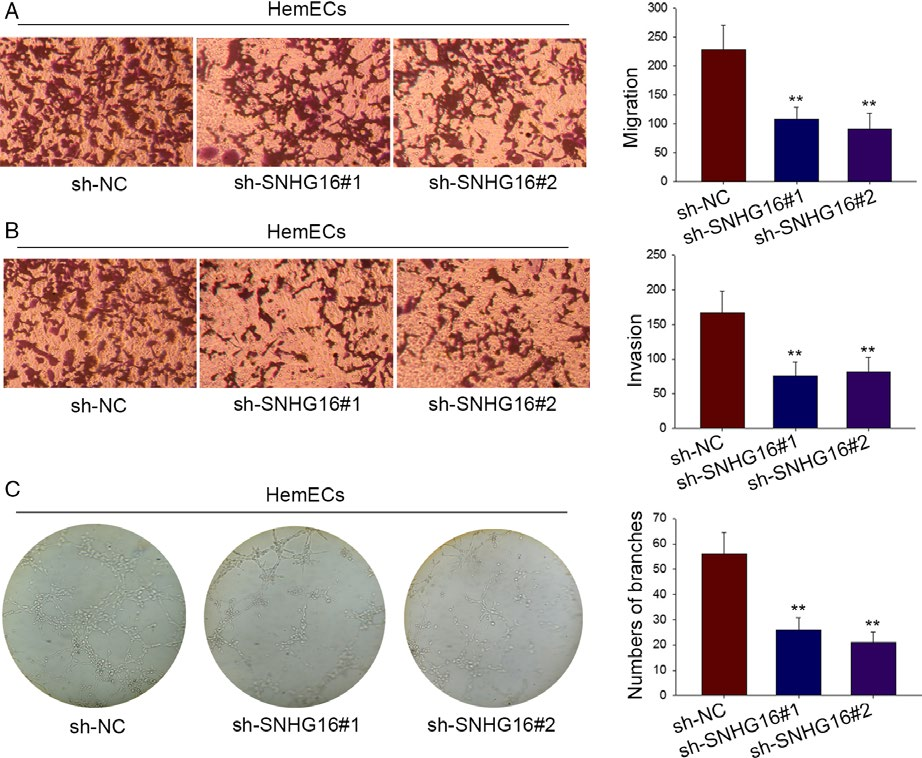
The influences of silenced SNHG16 on migration, invasion, and vasoformation of HemECs cells. A‐B, Transwell chamber was utilized for analyzing the change of invasion and migration in HemECs when SNHG16 expression was silenced. C, Tube formation assay assisted us of proving effects of sh‐SNHG16 on tube formation of HemECs

Transwell chamber was utilized to test invasion and migration of HemECs cells after SNHG16 was downexpressed. Obviously, silenced SNHG16 suppressed cell migration and invasion (Figure 2A,B). Finally, the effects of sh‐SNHG16 on tubule formation were verified. The result manifested that the complicated and ramified network of tubules in HemECs cell was changed to a simple one after SNHG16 was silenced (Figure 2C).

### 3.3 SNHG16 can bind with miR‐520d‐3p in HemECs

**Figure 3:**
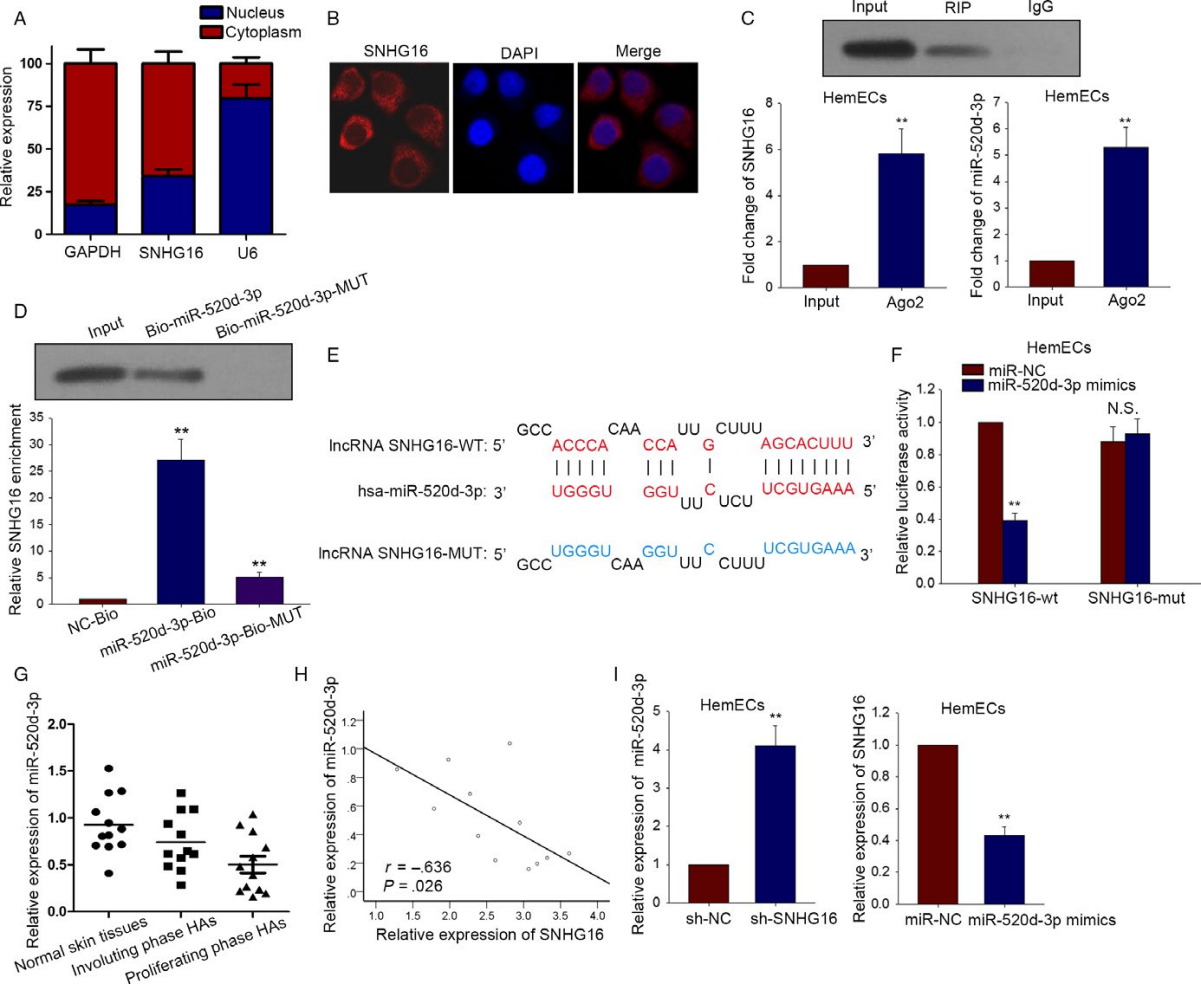
SNHG16 can bind with miR‐520d‐3p in HemECs. A, Subcellular fractionation assay examined the localization of SNHG16 in HemECs cell. B, RNA FISH was performed to probe the localization of SNHG16 in HemECs cell. C, RIP assay was conducted in HemECs with antibodies against Ago2 to find the combination between SNHG16 and miR‐520d‐3p. D, The binding relation between SNHG16 and miR‐520d‐3p was verified by pull‐down assay. E, The binding sites were obtained between two types of SNHG16 (wild type and mutant type) and miR‐520d‐3p. F, Dual‐luciferase assay was also performed to confirm the interaction between SNHG16 and miR‐520d‐3p. G, The expression of miR‐520d‐3p was tested in three different types of tissues. H, The correlation between SNHG16 and miR‐520d‐3p was proved by Spearman's correlation method. I, qRT‐PCR examined the effect of silenced SNHG16 on miR‐520d‐3p expression and the impact overexpressed miR‐520d‐3p on SNHG16 expression. Data were acquired from multiple experiments for mean ± SD. *P < .05, **P < .01 compared with controls

Although SNHG16 has been reported to be a ceRNA in human cervical cancer,^15^ the underlying molecular mechanism of it in hemangioma remains not explored. Here, we supposed that SNHG16 acted as a ceRNA in HemECs cells. First of all, subcellular fractionation assay was carried out in HemECs cell to identify the localization of SNHG16 (Figure 3A). As a result, SNHG16 was located in cytoplasm of HemECs cell. To acquire the adequate evidence, RNA FISH was also conducted to detect the localization of SNHG16 (Figure 3B). Similarly, SNHG16 was found to be located in cytoplasm of HemECs cell. Next, we found out 64 miRNAs which could bind with SNHG16 from StarBase database (http://www.lncrnablog.com/starbase-v2-0-for-decoding-rna-interaction-networks/). In order to make further confirmation, we silenced SNHG16 to measure the relative expression levels of all those 64 miRNAs (Table S1). As a consequence, the highest expression was shown in miR‐520d‐3p. Therefore, miR‐520d‐3p was chose to be the target gene for next experiments. RIP assay was conducted in HemECs with Ago2 antibody (Figure 3C). The results displayed that both SNHG16 and miR‐520d‐3p tended to be enriched in Ago2‐containing beads compared with that of control IgG. The result of pull‐down assay revealed that SNHG16 could be pulled down by bio‐miR‐520‐3p‐WT, while it could not be pulled down by bio‐miR‐520‐3p‐MUT and Bio‐NC (Figure 3D). As displayed in Figure 3E, we discovered that only the wild‐type SNHG16 could bind with miR‐520d‐3p. Then, dual‐luciferase assay was also performed to confirm the interaction between SNHG16 and miR‐520d‐3p. Seen from Figure 3F, we uncovered that only the luciferase activity of the wild‐type SNHG16 could be cut down by miR‐520d‐3p mimics. As displayed in Figure 3G, the lowest expression of miR‐520d‐3p was detected in proliferating phase HA tissues. Spearman's correlation analysis helped find the negative correlation between SNHG16 and miR‐520d‐3p in proliferating phase HA tissues (Figure 3H). Finally, qRT‐PCR was applied to make further confirmation about the relation between SNHG16 and miR‐520d‐3p in HemECs cell (Figure 3I). Silenced SNHG16 was able to largely increase miR‐520d‐3p expression. However, overexpressed miR‐520d‐3p caused the inhibition of SNHG16 expression. Therefore, we could conclude that SNHG16 might acted as a ceRNA to bind with miR‐520d‐3p.

### 3.4 SNHG16 regulates STAT3 through binding with miR‐520d‐3p

**Figure 4:**
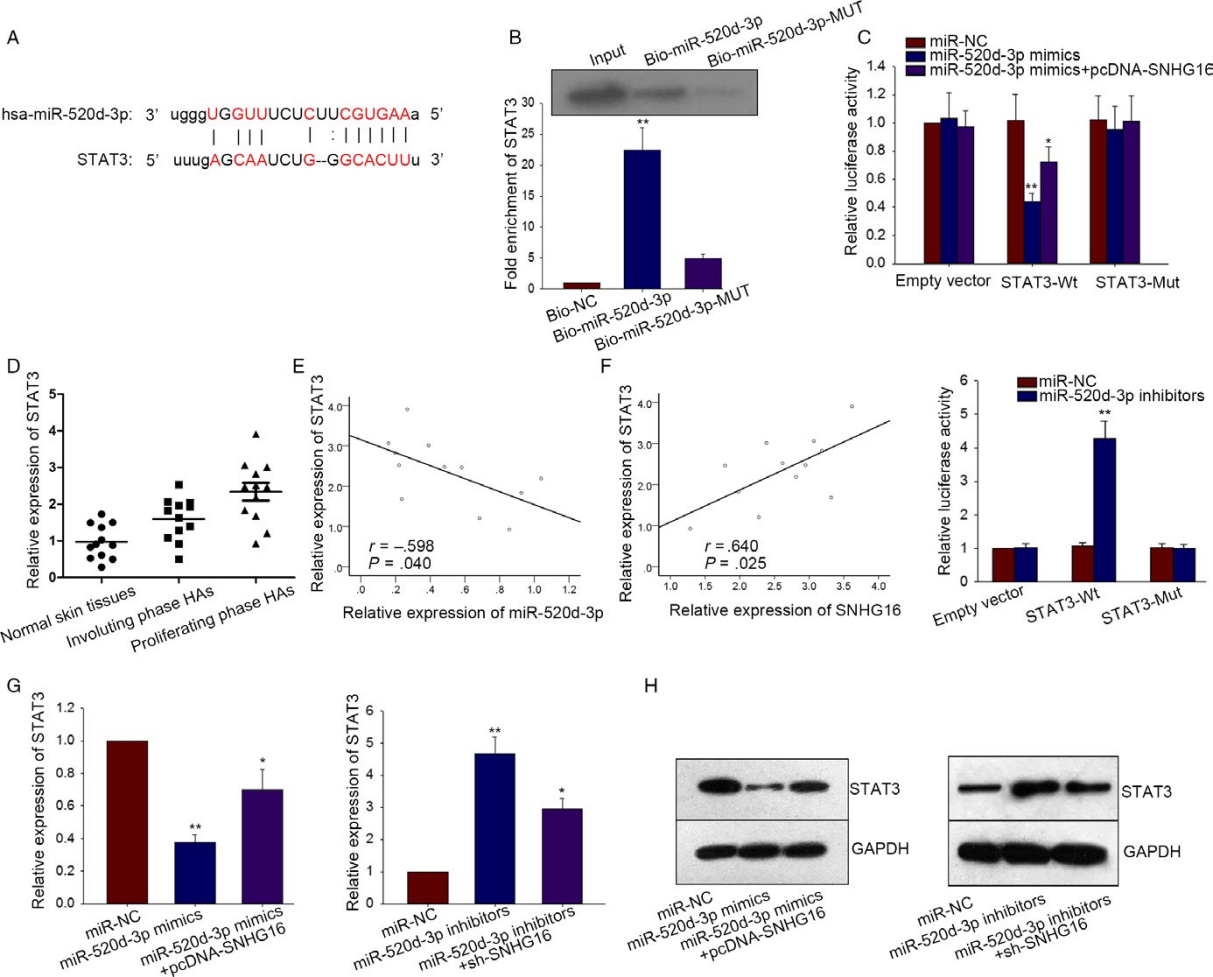
SNHG16 regulates STAT3 through binding with miR‐520d‐3p. A, The binding sites between miR‐520d‐3p and STAT3 were obtained by means of bioinformatics analysis. B, Pull‐down assay was conducted in HemECs cell to demonstrate the relation between miR‐520d‐3p and STAT3. C, Dual‐luciferase reporter assay showed an obvious result that only the luciferase activity of wild‐type STAT3 could be affected by miR‐520d‐3p mimics/inhibitors. D, The expression of STAT3 was detected in three types of tissues. E‐F, The correlation between miR‐520d‐3p and STAT3 as well as the correlation between SNHG16 and STAT3 was proved by Spearman's correlation method. G‐H, miR‐520d‐3p mimics and pcDNA‐SNHG16 as well as sh‐SNHG16 and miR‐520d‐3p inhibitors were transfected into HemECs to detect the corresponding changes taken place in mRNA level and protein level of STAT3. Data were acquired from multiple experiments for mean ± SD. *P < .05, **P < .01 compared with controls

As we thought SNHG16 acted as a ceRNA in HemECs, we found out a mRNA and obtained its binding sites with miR‐520d‐3p from a website. (http://34.236.212.39/microrna/getMrna.do?gene=6774&utr=11493&organism=9606) (Figure 4A). Likewise, the result of pull‐down assay revealed that STAT3 could be pulled down by bio‐miR‐520‐3p‐WT, while it could not be pulled down by bio‐miR‐520‐3p‐MUT and Bio‐NC (Figure 4B). To obtain further confirmation, dual‐luciferase reporter assay was designed and conducted. As displayed in Figure 4C, only the luciferase activity of wild‐type STAT3 could be reduced by miR‐520d mimics and was recovered again by treating with pcDNA‐SNHG16. The luciferase activity of mutant type or empty vector was almost not changed. This could prove that STAT3 bond with miR‐520d‐3p and was positively modulated by SNHG16. To acquire further evidence, the luciferase activity of STAT3 was detected after miR‐520d‐3p was silenced (Figure 4C). As a result, the decreased luciferase activity was observed in STAT3‐WT. The highest expression of STAT3 was tested in proliferating phase HA tissues (Figure 4D). The negative correlation between STAT3 and miR‐520d‐3p as well as the positive correlation between SNHG16 and STAT3 was analyzed (Figure 4E,F). To obtain final proof, sh‐SNHG16 and miR‐520d‐3p inhibitors were separately transfected into HemECs cells to detect the changes took place in mRNA level and protein level of STAT3. Obviously, both mRNA level and protein level of STAT3 were reduced by transfecting with miR‐520d‐3p mimics, while they were strengthened by treating pcDNA‐SNHG16 (Figure 4G,H). Moreover, the increased mRNA level and protein level of STAT3 were observed after miR‐520d‐3p was silenced, while both levels were decreased again by transfecting of sh‐SNHG16 (Figure 4G,H). Therefore, we could conclude that SNHG16 positively affect STAT3 expression in HemECs cells through sequestering miR‐520d‐3p.

### 3.5 The effects of SNHG6‐miR‐520d‐3p‐STAT3 pathway on HemECs cell activities

**Figure 5:**
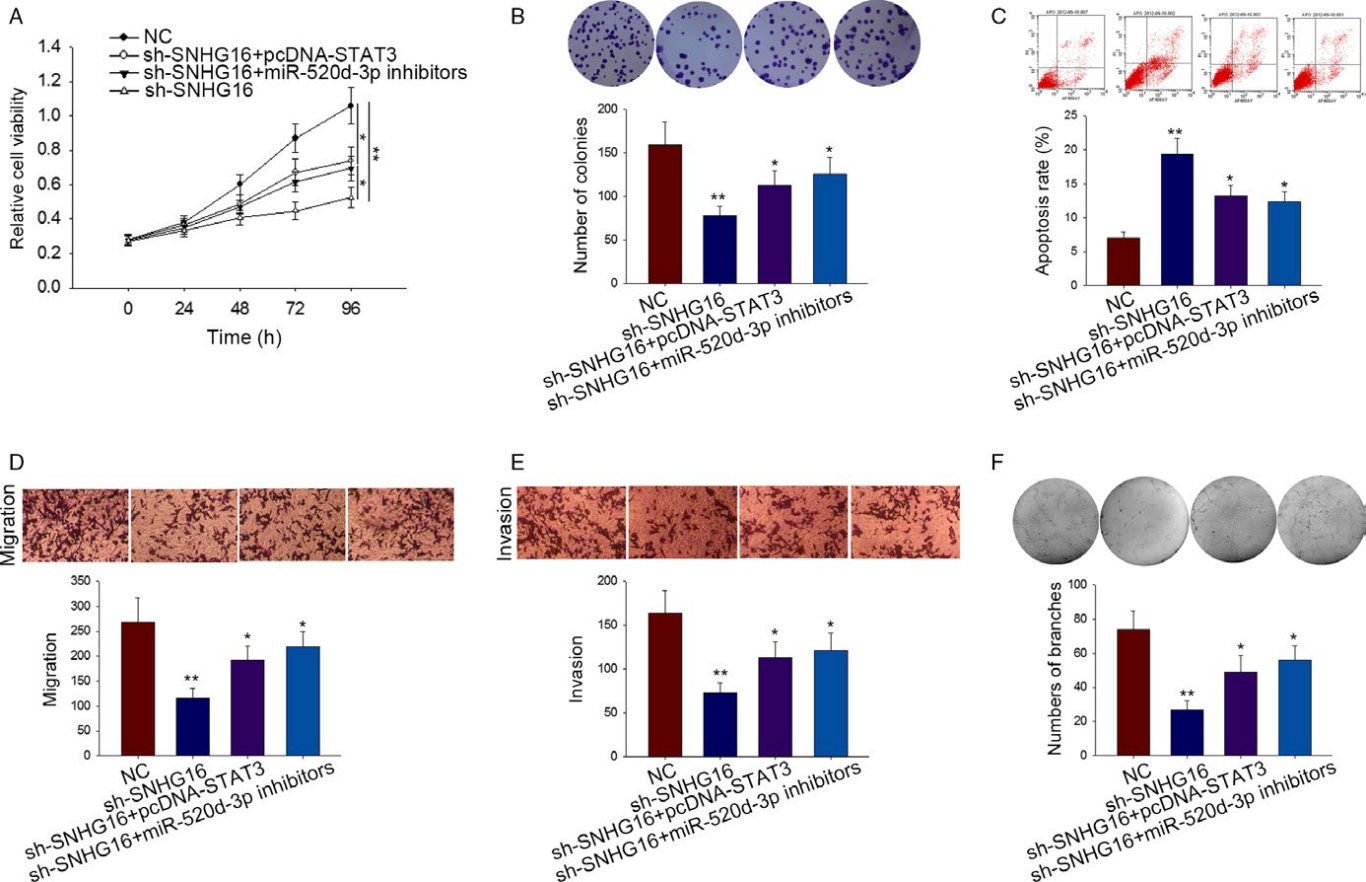
The effects of SNHG6‐miR‐520d‐3p‐STAT3 pathway on HemECs cell activities. A‐B, The proliferation ability of indicated HemECs was detected by MTT and colony formation assays. C, The apoptosis of transfected HemECs cell was detected by flow cytometry analysis. D‐E, The migration and invasion of HemECs cells were detected using transwell assay. F, Tube formation assay was carried out in indicated HemECs cells to investigate the vasoformation. Data were acquired from multiple experiments for mean ± SD. *P < .05, **P < .01 compared with controls

In order to know the actual effects of SNHG6‐miR‐520d‐3p‐STAT3 axis on HemECs cell activities, rescue assays were designed and carried out in HemECs cells. Firstly, the proliferation ability of HemECs was detected by MTT and colony formation assays. We found the decreased proliferation of SNHG16‐downregulated HemECs cell was strengthened by separately transfecting with pcDNA‐STAT3 and miR‐520d‐3p inhibitors (Figure 5A,B). The result of flow cytometry analysis suggested that increased cell apoptosis caused by sh‐SNHG16 was rescued by pcDNA‐STAT3 and miR‐520d‐3p inhibitors (Figure 5C). The same transfection was conducted in HemECs to detect migration and invasion abilities through performing transwell assay. The results were consistent with MTT assay (Figure 5D,E). The results of tube formation assays showed the inhibitory effects of sh‐SNHG16 on vasoformation were recovered by pcDNA‐STAT3 and miR‐520d‐3p inhibitors (Figure 5F).

## 4 DISCUSSION

Previous studies have demonstrated that lncRNA SNHG16 acts as a motivator in various human tumors, such as breast cancer,^19^ NSCLC,^20^ and colorectal cancer.^21^ It is also reported that SNHG16 participated in various biological activities, especially cell proliferation, invasion, and migration. In this study, we tried to deeply study the impacts of SNHG16 on cell activities of HemECs. ceRNA pattern is a very important biological pathway in which miRNAs‐mediated lncRNA could participate in the whole progress of tumors via ceRNA regulatory mechanisms. Many researches have provided the valid evidence to demonstrate the great effects of lncRNAs on complex processes of human diseases.^22^, ^23^, ^24^, ^25^ As diagnostic or prognostic markers, ceRNAs have been uncovered in many reports.^26^, ^27^, ^28^, ^29^, ^30^, ^31^, ^32^ In some cases, lncRNAs can participate in ceRNA pattern to modulate gene expression.^33^, ^34^ In this study, we supposed that SNHG16 might be a ceRNA in HemECs.

MiRNAs had been verified to be independent regulators in many cancers. They also have some particular role in ceRNA axis. miRNAs are usually taken as the target genes of lncRNAs to modulate tumorigenesis. They participated in many biological processes of human tumors through interacting with lncRNAs or target mRNAs. miR‐129‐5p participated in a ceRNA pathway in colon cancer.^35^ miR‐181a‐5p and multiple myeloma progression.^36^ All above examples showed the interaction between miRNAs and mRNA in cancers.

Our findings showed a regulatory model of ceRNA (SNHG16‐miR‐520d‐3p‐STAT3). Then, we began to do our research. We first silenced SNHG16 to detect cell activities (proliferation, migration, and invasion) in HemECs. Loss‐of‐function assay reflected a phenomenon that silenced SNHG16 greatly suppressed proliferation, metastasis, and vasoformation of HemECs. The potential molecular mechanisms of SNHG16 were further probed in HemECs. It was detected to be located in cytoplasm of HrmECs. To certify this hypothesis, we performed RIP assay and pull‐down assay, the result also indicated that SNHG16 could bind with miR‐520d‐3p. The binding sites between SNHG16 and miR‐520d‐3p were searched through using bioinformatics analysis. Similarly, the binding sites between miR‐520d‐3p and STAT3 were obtained. In order to clearly elucidate the combination among these three genes, dual‐luciferase reporter assay was utilized. We found SNHG16 positively modulated STAT3 expression by sequestering miR‐520d‐3p. At length, rescue assays were carried out to prove the effects of SNHG16‐miR‐520d‐3p‐STAT3 on proliferation, apoptosis, metastasis, and vasoformation of HemECs. The whole study aimed to explore novel ceRNA pathway in HemECs. This study was not further detected the upstream molecular mechanism of SNHG16‐miR‐520d‐3p‐STAT3 axis. Therefore, we will do further study in the future. We thought this study would be helpful for investigating more biological pathway for endothelial cells research in hemangioma.

## CONFLICT OF INTEREST

None declared.
